# The impact of visual display of human motion on observers’ perception of music performance

**DOI:** 10.1371/journal.pone.0281755

**Published:** 2023-03-08

**Authors:** Nádia Moura, Pedro Fonseca, Márcio Goethel, Patrícia Oliveira-Silva, João Paulo Vilas-Boas, Sofia Serra

**Affiliations:** 1 School of Arts, Research Centre in Science and Technology of the Arts, Universidade Católica Portuguesa, Porto, Portugal; 2 Porto Biomechanics Laboratory, Faculty of Sport, University of Porto, Porto, Portugal; 3 Centre of Research, Education, Innovation and Intervention in Sport, Faculty of Sport, University of Porto, Porto, Portugal; 4 Human Neurobehavioral Laboratory, Research Centre for Human Development, Universidade Católica Portuguesa, Porto, Portugal; Federal University of Paraiba, BRAZIL

## Abstract

In investigating the influence of body movement in multimodal perception, human motion displays are frequently used as a means of visual standardization and control of external confounders. However, no principle is established regarding the selection of an adequate display for specific study purposes. The aim of this study was to evaluate the effects of adopting 4 visual displays (point-light, stick figure, body mass, skeleton) on the observers’ perception of music performances in 2 expressive conditions (immobile, projected expressiveness). Two hundred eleven participants rated 8 audio-visual samples in expressiveness, match between movement and music, and overall evaluation. The results revealed significant isolated main effects of visual display and expressive condition on the observers’ ratings (in both, *p* < 0.001), and interaction effects between the two factors (*p* < 0.001). Displays closer to a human form (mostly skeleton, sometimes body mass) exponentiated the evaluations of expressiveness and music-movement match in the projected expressiveness condition, and of overall evaluation in the immobile condition; the opposite trend occurred with the simplified motion display (stick figure). Projected expressiveness performances were higher rated than immobile performances. Although the expressive conditions remained distinguishable across displays, the more complex ones potentiated the attribution of subjective qualities. We underline the importance of considering the variable display as an influencing factor in perceptual studies.

## Introduction

Audience perception of performative arts is highly shaped by their visual component. A substantial amount of information can be conveyed through the performers’ body behaviour, including expressive [1–4] and emotional intentions [5–8]. For example, actors and musicians may reinforce verbal speech and musical expression with gestures. Body movements are also reported to accompany structural features of musical pieces [1, 9, 10], thus modifying the auditory perception of spectators [3, 11]. Briefly, body motion has the potential to enrich artistic experiences, bringing together performers and audience in meaningful interactions [12, 13]. The understanding of this phenomenon has motivated a corpus of perceptual studies which imply the presentation of biological motion accurately enough to identify subtle differences, but also standardized enough to narrow the attention to the motion itself. It has been demonstrated that factors like the observers’ level of expertise [14, 15], or the performers’ gender [16] and motion type [3, 4, 17] influence the judgement of observers, but little data is found concerning the impact of the type of visual display adopted to present the stimuli, or the criteria used in its selection.

Within the broader scope of biologic motion perception, four display types are commonly used: traditional video footage [3, 6, 16, 18–20], point-light displays [21–33], stick figures [4, 14, 34–39], and varied animated characters [24, 32, 40–44]. Although regular video provides more ecological, realistic representations [18], the other displays enable the de-characterization of the subject, allowing for a better control of extraneous characteristics like gender [16] or dress style [20, 45].

Introduced by Johansson [21, 22], the point-light display (PL) was the first simplified version of human motion used in perception studies of human gait. It turned out to be impressively easy to perceive, given that less than 200 milliseconds and 10 dots were enough for a person to recognize an articulated human shape [22]. PL further revealed to be successful in the identification of individuals [26, 46], genders [27, 28, 47] and emotions [48, 49]. In music performance, it was introduced in a series of pioneer movement studies with musicians for detecting different levels of expressivity [29–31]. Recently, PL has been used for studying individual differences of the observers [23], impairments associated with autism [25] and kinaesthetic experiences in virtual reality (VR) [32]. Stick figures and volumetric models eventually emerged from the need to represent body segments and create more realistic representations [50]. Currently, no convention is established about which displays to adopt for perceptual evaluation, and it is possible to find studies integrating either PL, stick figures and animated characters in the last two years [e.g., 23, 39, 44].

Two opposite perspectives were extracted from the existing literature about this topic. The first supports that distinct display conditions do not elicit significant differences in perceptual tasks [18, 32, 34, 40]. In fact, similar accuracy results among participants are reported in: person identification, when comparing video with PL [18]; motion similarity assessment in different perceptual spaces and metrics, when comparing PL with stick figures [34]; and in emotion recognition, when comparing video with a humanoid avatar [40, 42]. Complementarily, in a VR study, the kinaesthetic experience of embodying self-avatars was as effective with virtual point-light body parts as with realistically animated ones [32].

The second perspective underpins that motion configurations do impact perceptual judgement, and that the ones most proximal to an anthropomorphic form convey better information than simplified versions [24, 35, 38, 48, 51]. Hodgins and colleagues [35] found that the detection of motion changes was better with an animated human polygonal model than with a stick figure. Increased accuracy in emotion recognition was reported by Atkinson and colleagues [48] in video stimuli when compared to PL, and by Carreno-Medrano and colleagues [38] in stick figures compared to motion trajectories only. Narang and colleagues [24] found that self-recognition of motion was better with realistic 3D avatars than PL, while motion recognition of others was better with PL. Further, a recent VR study by Shin and colleagues [51] compared the effect of a hand-shaped avatar, a non-hand shaped avatar and no avatar in a motor learning task, confirming that the learning rate was facilitated by recognizing one’s body form. This perspective is also reinforced by multiple studies in the field of human-computer interaction, where it is well established that animated characters influence how people engage with and perceive virtual environments [41, 52–54].

The purpose of this study was to investigate the effect of the visual display on the observers’ perception of music performances, in order to build a robust foundation for the design of further perceptual tests. We expanded on previous studies by comparing four display types (PL: point-light, SF: stick figure, BM: body mass, SK: skeleton) in two performative expressive conditions (IMO: immobile, EXP: projected expressiveness). In the task, participants were asked to score each recording in terms of expressiveness, match between movement and music, and overall evaluation. We expected that displays proximal to an anthropomorphic form (BM and SK) would score higher than the simplified ones (PL and SF), and that recordings in EXP condition would have higher ratings than in IMO condition.

## Materials and methods

### Participants

A total of 211 participants took part in this study, recruited by email and social media within the contact network of the authors. The inclusion criteria were: being over 18 years old, healthy and conscious; having normal or normal-to-corrected vision and hearing; having a computer, tablet or smartphone, and headphones or speakers. In the recruitment sheet, a disclaimer was included informing that both musicians and non-musicians were eligible.

The gender distribution was 112 (53.1%) females, 98 (46.4%) males, and 1 (0.5%) participant preferred not to say. The participants’ age was distributed as: 72 (34.1%) between 25–34 years old; 57 (27.0%) between 18–24 years old, 36 (17.1%) between 35–44 years old, 27 (12.8%) between 45–54 years old, 15 (7.1%) between 55–64 years old, 3 (1.4%) more than 65 years old, and 1 (0.5%) preferred not to say. Regarding the country where they have lived the longest, 160 (75.8%) participants stated Portugal, 19 (9.0%) England, 5 (2.4%) Brazil, 4 (1.9%) France, 3 (1.4%) Spain, 3 (1.4%) Scotland, and the other 17 (8.1%) were residually distributed by other countries. Concerning educational background, 74 (35.1%) participants had completed a Bachelor’s Degree, 73 (34.6%) a Masters’ Degree, 44 (20.9%) High School or less, 11 (5.2%) PhD or higher, 7 (3.3%) technical course, and 2 (0.9%) preferred not to say. In relation to musical background, 80 (37.9%) reported to have had general music lessons in regular school, 53 (25.1%) attended a music school or conservatoire, 34 (16.1%) attended higher education in Music, 32 (15.2%) never had music classes nor musical practice, 8 (3.8%) answered “Other”, and 4 (1.9%) never had music classes but are amateur musicians.

Ethical approval for this study was given by the Ethical Committee for Health of the Portuguese Catholic University (CES-UCP), with the protocol number 196/2022. Participants gave their informed consent, written in agreement with the Declaration of Helsinki (2013).

### Stimuli

The base stimuli used for this study were two motion and audio recordings of one performer playing a fragment (bars 98–103) from the Saxophone Concerto in E-flat Major by Glazunov [55]. One recording corresponded to immobile condition (IMO), and the other to projected expressiveness condition (EXP). In the IMO condition, the performer was instructed to perform with the least amount of movement possible, restricted to the essential movements for effective sound production, whereas in the EXP condition, the performer was instructed to move as projected for a public performance, with no restrictions to expressive movement. These conditions were retrieved from referential literature in the field [e.g., 29, 56]. In the recording, the performer was in the final year of the bachelor’s degree in Classical Saxophone Performance and had 13 years of classical saxophone training.

**Fig 1:**
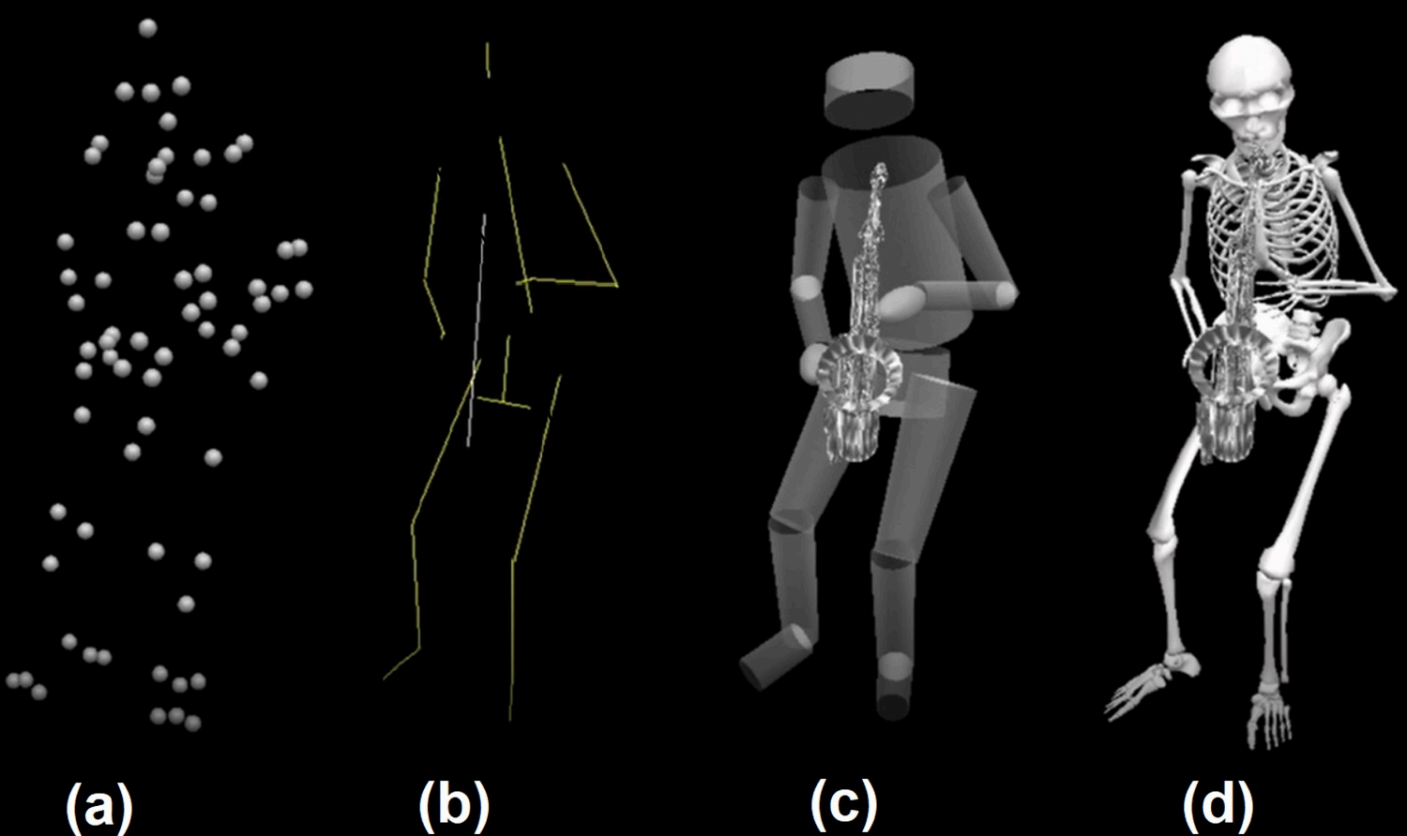
The four types of visual display used in this study. (a) PL: point-light, (b) SF: stick figure, (c) BM: body mass and (d) SK: skeleton.

The motion files were processed through Visual3D v6 (C-Motion Inc., USA) to generate visualizations in the 4 visual displays presented in Fig 1. We then used Open Broadcaster Software 27.2.3 (OBS Project) to video record each motion file in each display, and DaVinci Resolve 17 (Blackmagic Design Pty Ltd., Australia) to synchronize the videos with the original audio tracks. For each expressive condition, the audio from the corresponding performance was used. This resulted in a total of 8 videos with 17 seconds of duration each (S1–S8 Videos) for assessment (2 expressive conditions x 4 visual displays).

### Procedure

The experiment was conducted as an online survey via Qualtrics platform (www.qualtrics.com). The survey was designed to be compatible with all portable devices so that a higher number of participations was achieved. Research shows that respondents are as likely to provide conscientious answers on smartphones as well as on computers, as long as the question layouts adapted to the dimensions of smaller screens [57]. For that, we used square videos to uniformize the visualization in portrait and landscape orientations and tested the survey in multiple devices and operating systems. The data collection period lasted 28 days (January 31^st^ to February 27^th^, 2022).

Prior to starting, an information sheet was presented to the participants including a description of the study, instructions for participation, as well as a remark about the voluntary nature of participation and the possibility of withdrawal at any time with no prejudice. Followingly, participants gave their written informed consent. The survey was organized as follows: sociodemographic group, stimuli group and one final optional question requesting a free comment about the experiment. Debriefing and contacts were presented in the last page.

In the stimuli group, audio-visual clips were randomized and presented in individual pages. They could be replayed as many times as wanted. To ensure participants watched the full clip at least once, a 20 second timer counting from the first click was set until the follow-up arrow appeared. Below the clip, three horizontal rating sliders were presented, corresponding to the scales adapted from the study conducted by Weiss and colleagues [4]: expressiveness (0 –Not at all expressive to 100 –Extremely expressive), match between movement and music (0 –Extremely disconnected to 100 –Extremely well matched), and overall evaluation (0 –Awful to 100 –Excellent). Participants gave three ratings per stimulus, corresponding to each assessment parameter. We used a scale with end labels (0–100), and the initial handle was placed at the middle (without numeric indication), to avoid the response bias caused by the initial positioning of the handle to the left-hand of the scale [58–60]. Numeric feedback was provided along with the movement of the handle. All rating responses were mandatory, and at least one click in each slider was needed to record the rating.

### Data analysis

#### Performance ratings

Before running the statistical tests, the assumptions of normal distribution for the variables of interest were tested. As a result, the data met the assumption of linearity and displayed no multicollinearity as well as no significant multivariate outliers, as assessed by Mahalanobis distance (*p* > 0.001). However, due to some violations of normality, as evaluated by Shapiro-Wilk’s test (*p* < 0.05), log, square-root, and box-cox transformations were tried on the raw data. Unfortunately, these transformations did not allow the normalization of the dataset. However, based on the Central Limit Theorem, which asserts that the sum of non-normally distributed observations approaches a normal distribution as the number of observations increases, namely if the sample size has 100 or more observations [61, 62], we considered that normality was not a significant issue for this dataset. In other words, considering the large sample size and the same result pattern using univariate nonparametric tests (as seen after running the Friedman 2-way ANOVAs for the main effect results, reported in S1 File), we considered it plausible to relax the assumptions and opted for using the parametric options applied to the raw data.

A two-way repeated measures multivariate analysis of variance (MANOVA) was performed on the mean rating scores of expressiveness (E), match between movement and music (MM), and overall evaluation (EV) with visual display (4 levels: PL, SF, BM, SK) and expressive condition (2 levels: IMO, EXP) entered as factors. When the MANOVA and subsequent univariate analyzes of variance (ANOVA) were statistically significant, differences were accessed using post-hoc Bonferroni corrected pairwise comparisons. The level of significance for all tests was set at α *=* 0.05. These analyses were performed using the SPSS 28 package (IBM SPSS Statistics, USA). To determine the observed power, post-hoc power analyses were conducted using G*Power 3.1.9.7. (G*Power, Germany), and the results are expressed as power percentages–PW (Power = (1 - β err prob) x 100).

#### Qualitative feedback

The qualitative data from the open comment question (*“Would you like to make a comment about the experiment*?*”*) was analyzed by means of content analysis. This method is used to determine the presence and frequency of terms, narratives and concepts within qualitative visual and textual datasets [63]. Firstly, the 18 voluntary responses were listed in Microsoft Excel and repeatedly read for familiarization. Then, they were organized into description units, categorized and counted according to 3 inducted categories (see Results).

## Results

### Main effects of display type and expressive condition

The results showed a significant main effect of the factors display, Pillai’s Trace = 0.092, *F* (9,1890) *=* 6.67, *p <* 0.001, and expressive condition, Pillai’s Trace = 0.736, *F*(3,208) *=* 192.86, *p <* 0.001, on the observers’ ratings. Descriptive statistics and follow-up pairwise comparisons (α = 0.05, Bonferroni corrected) by display and expressive condition are presented in Tables 1 and 2, which should be read by crossing rows with columns. For example, in Table 1, fourth row SK does not show significant differences from PL and shows a positive difference from SF and BM.

**Table 1:** Descriptive statistics and pairwise comparisons of each dependent variable across display conditions.

Rating Parameters	Display Condition	*M*	*SE*	95% Confidence Interval		Pairwise Comparisons (*Row vs Column*)		
				Low	High	PL	SF	BM
Expressiveness	PL	47.74	1.53	44.72	50.75			
	SF	45.9	1.57	42.8	48.99	=		
	BM	46.59	1.52	43.6	49.57	=	=	
	SK	49.99	1.49	47.05	52.93	=	>>	>>
Match between movement and music	PL	45.72	1.27	43.23	48.21			
	SF	44.61	1.32	42.01	47.21	=		
	BM	47.61	1.21	45.23	50	=	>	
	SK	48.14	1.31	45.55	50.72	>	>>	=
Overall evaluation	PL	52.2	1.39	49.46	54.94			
	SF	50.17	1.44	47.34	53.01	<<		
	BM	52.63	1.29	50.08	55.18	=	>	
	SK	55.41	1.33	52.79	58.02	>>	>>	>

Positive difference: >> (*p* < 0.01); > (*p* < 0.05). Negative difference: << (*p* < 0.01).

Not significant: = (*p* ≥ 0.05).

***M***: Mean; ***SE***: Standard Error; **PL**: Point-light; **SF**: Stick Figure; **BM**: Body Mass; **SK**: Skeleton.

**Table 2:** Descriptive statistics and pairwise comparisons of each dependent variable across expressive conditions.

Rating Parameters	Expressive Condition	*M*	*SE*	95% Confidence Interval		Pairwise Comparisons (*Row vs Column*)
				Low	High	IMO
Expressiveness	EXP	61.68	1.42	58.88	64.48	>>
	IMO	33.42	1.81	29.86	36.99	
Match between movement and music	EXP	65.61	1.33	62.99	68.22	>>
	IMO	27.43	1.45	24.57	30.3	
Overall evaluation	EXP	66.71	1.31	64.13	69.29	>>
	IMO	38.5	1.61	35.33	41.67	

Positive difference: >> (*p* < 0.01).

***M***: Mean; ***SE***: Standard Error; **IMO**: Immobile; **EXP**: Projected Expressiveness.

**Fig 2:**
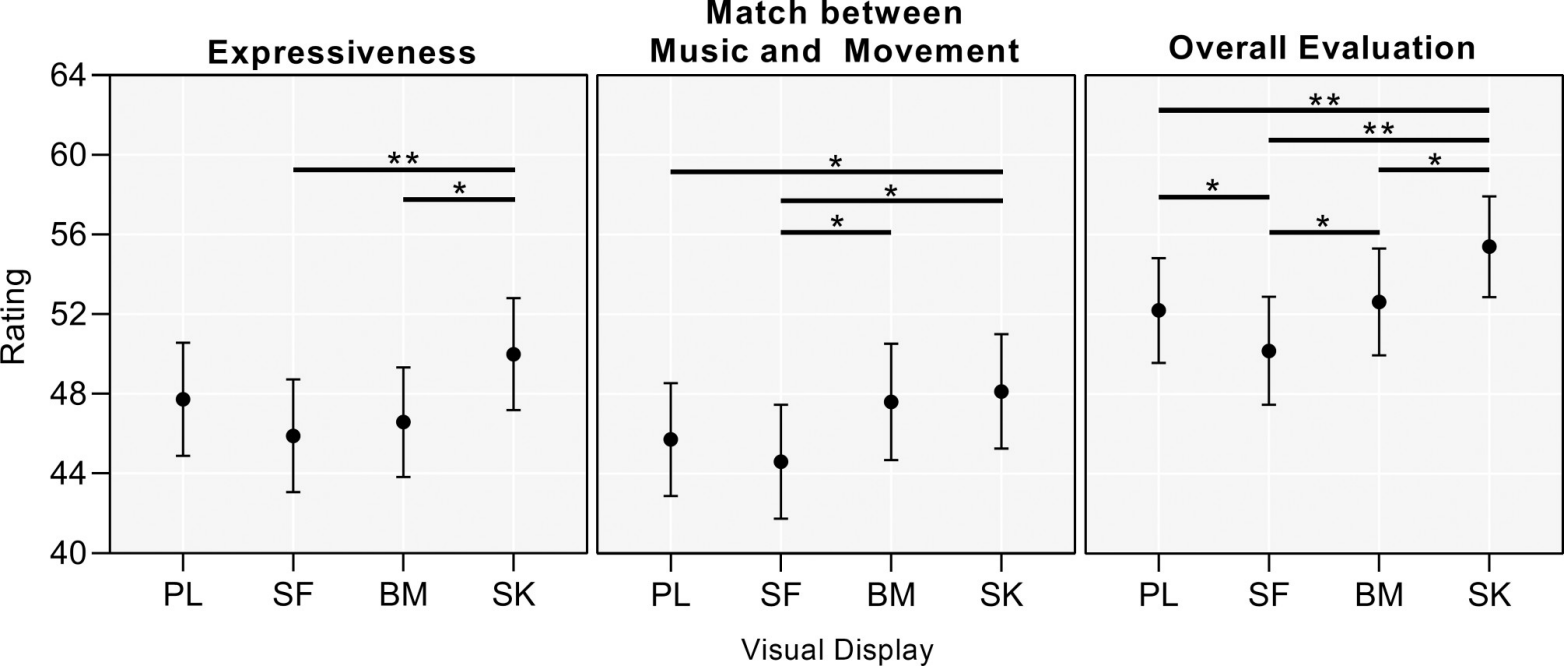
Mean ratings (with 95% confidence intervals) and significant differences between the four visual displays. PL: point-light; SF: stick-figure; BM: body-mass; SK: skeleton; * stands for *p* < 0.05; ** stands for *p* < 0.01.

In the factor display, for the expressiveness parameter, SK (49.99 ± 29.33) scored significantly higher than BM (46.59 ± 28.73) (*p =* 0.001, Δ = 3.41) and SF (45.9 ± 29.61) (*p <* 0.001, Δ = 4.1); the significance in relation to PL (47.74 ± 29.59) was almost reached (*p =* 0.051, Δ = 2.26). For the match between music and movement parameter, SK (48.14 ± 29.9) scored significantly higher than SF (44.61 ± 29.87) (*p =* 0.001, Δ = 3.524) and PL (45.72 ± 29.48) (*p =* 0.047, Δ = 2.415); additionally, BM (47.61 ± 30.51) scored significantly higher than SF (*p =* 0.012, Δ = 3.002). Regarding the overall evaluation parameter, SK (55.41 ± 26.4) scored significantly higher than PL (52.2 ± 27.38) (*p <* 0.001, Δ = 3.206), than SF (50.17 ± 28.29) (*p <* 0.001, Δ = 5.235) and than BM (52.63 ± 27.92) (*p =* 0.017, Δ = 2.78); and SF scored significantly lower than BM (*p =* 0.017, Δ = -2.455) and than PL (*p =* 0.011, Δ = -2.028). The post-hoc observed power for all comparisons was higher than 99%, except for the PL-BM comparison in the overall evaluation rating, where it was 90.76%. For a graphical representation of these findings, see Fig 2.

In the factor expressive condition, EXP scored significantly higher than IMO in all three parameters of rating: expressiveness (respectively, 61.68 ± 25.52, 33.42 ± 27.78; *p <* 0.001, Δ = 28.257), match between movement and music (respectively, 65.61 ± 23.13, 27.43 ± 23.17; *p <* 0.001, Δ = 38.173) and overall evaluation (respectively, 66.71 ± 21.89, 38.5 ± 25.36; *p <* 0.001, Δ = 28.212). The post-hoc observed power for all comparisons was 100%.

### Interaction effects

**Fig 3:**
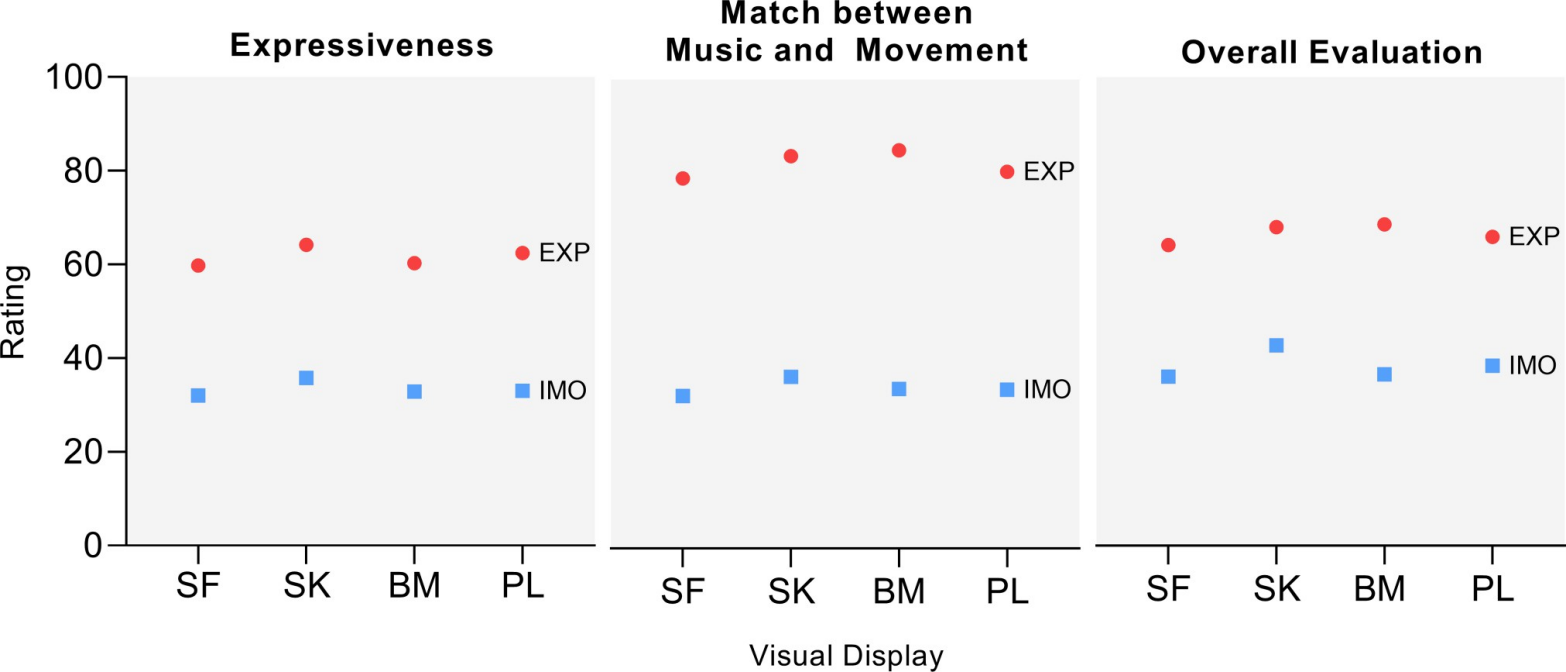
Interaction plots of the mean ratings of the visual displays in EXP and IMO conditions. EXP: projected expressiveness; IMO: immobile; PL: point-light; SF: stick-figure; BM: body-mass; SK: skeleton.

There was a significant interaction effect between visual display and expressive condition, *F* (9,1890) *=* 4.72, *p <* 0.001, for the ratings, showing that the effect of the expressive condition was mediated by the visual display. Follow-up Bonferroni corrected pairwise comparisons were performed. For the expressiveness parameter, in the EXP condition, the SK (*M* = 64.22 ± 22.5) scored significantly higher than BM (*M* = 60.28 ± 23.5) (*p =* 0.003, Δ = 3.94) and SF (*M* = 59.79 ± 23.99) (*p <* 0.001, Δ = 4.44); in the IMO condition, the SK (*M* = 35.76 ± 28.45) scored significantly higher than SF only (*M* = 32.01 ± 28.15) (*p <* 0.001, Δ = 3.75). For the match between music and movement parameter, in the EXP condition, the SK (*M* = 66.97 ± 22.65) scored significantly higher than SF (*M* = 63.16 ± 23.85) (*p* = 0.009, Δ = 3.81), and BM (*M* = 67.98 ± 23.03) also scored significantly higher than SF (*p =* 0.026, Δ = 4.82); in the IMO condition, no significant differences were found. For the overall evaluation parameter, in the EXP condition, the SK (*M* = 68.04 ± 21.41) scored significantly higher than SF (*M* = 64.21 ± 23.12) (*p* < 0.001, Δ = 3.83) and the BM also scored significantly higher than SF (*M* = 68.63 ± 20.67) (*p =* 0.009, Δ = 4.42); in the IMO condition, the SK (*M* = 42.77 ± 24.83) scored significantly higher than PL (*M* = 38.45 ± 25.22) (*p* = 0.003, Δ = 4.33), BM (*M* = 36.63 ± 24.91) (*p* < 0.001, Δ = 6.14) and SF (*M* = 36.14 ± 25.94) (*p* < 0.001, Δ = 6.64), and SF scored significantly lower than PL (*p* = 0.004, Δ = -2.31). A graphical representation of the interactions is presented in Fig 3.

### Open comment question

Eighteen participants provided textual feedback through the voluntary open comment question, which was organized into the following content categories: movement (9 mentions), music (7 mentions) and opinions about the study (5 mentions). On the movement category, 5 participants considered the musician’s movement to be positive, mentioning it enhances the experience and makes it more enjoyable (one adding that the skeleton made motion perception easier), while other 4 considered movement to be unimportant for performance evaluation, as attention should be focused on the sound (2 mentioned movement may prejudice performance and one mentioned skilled musicians are less affected by this parameter). On the music category, 6 participants referred they perceived the audio as alike or the same throughout the samples, and one indicated to have mainly focused on sound for the assessment. On the category opinions about the study, 4 participants mentioned they considered the study interesting/creative, and one participant suggested expressivity is a complex concept to evaluate.

## Discussion

The results revealed that both factors display and expressive condition had significant main effects in the participants’ ratings. Further interaction effects were found suggesting that the expressive condition was mediated by the visual display in some cases. Although participants consistently rated projected expressiveness performances higher than immobile ones, certain displays exponentiated rating differences between the two conditions. Specifically, for expressiveness, SK rated significantly higher than BM and SF in the EXP condition, whereas it only rated higher than SF in the IMO condition. For match between movement and music, SK and BM rated significantly higher than SF in the EXP condition, whereas no significant differences were found in the IMO condition. In these scenarios, displays closer to a human form elicited higher ratings for the EXP condition. Nevertheless, a contrasting pattern was unveiled for overall evaluation: SK and BM rated higher than SF in EXP condition, whilst SK rated higher than all the other displays in the IMO condition. Here, displays closer to a human form elicited higher ratings for the IMO condition.

Generally, these findings align with previous literature where anthropomorphic-like displays aided the perception of motion features, when compared to reduced motion versions [24, 35, 38, 48, 51]. Atkinson and colleagues [48] found that emotion classification accuracy from body expression was improved in video in relation to PL, and that movement exaggeration exponentiated accuracy levels for some emotions. Similarly, motion sequences were better distinguished with a polygonal model than SF in Hodgins and colleagues [35], and emotional states were more effectively recognized from SF than trajectories alone in Carreno-Medrano and colleagues [38]. However, these studies also confirm that it is possible to perceive motion variations from reduced information, although success rate decreases [35, 38, 48]. On the other hand, in Narang and colleagues [24], self-recognition accuracy was higher for virtual avatars when compared to PL, but recognition of others was improved in PL. It seems, therefore, that the perceptual effect of visual display varies depending on the task. While some tasks may be equivalently executed with any display, others may be facilitated or prejudiced by that factor. In this sense, our results should not be extrapolated to say human-like displays perform better than reduced versions in conveying expressiveness. Instead, they confirm that there are significant differences between simplified and human-like displays, with the latter potentiating ratings of expressiveness and music-movement match in expressive movement conditions and overall evaluation in low movement conditions.

Apart from the anatomic detail provided by SK, it should also be highlighted that it is the only display embodying minimal facial features, which may have contributed to the attribution of the high-level properties identified in the results. Facial attributes provide essential information for identifying basic emotional expressions in others [64]. When occluded, recognition accuracy of such states is affected [65]. The fact that SK had identifiable face parts (mouth, nose, eyes), although fixed, may have led participants to associate it with expression, when compared to the other non-faced displays. It may also have promoted a higher level of empathy, potentially leading participants to score it more favorably. Empathic behaviors are influenced by the relationship between observer and target, field in which similarity is described as an effect factor [66]. This was a non-anticipated variable in our study, which deserves further exploration.

Contrarily, the lowest scores were regularly observed in SF display across rating parameters and expressive conditions. One possible explanation for this is that SF is a highly synthetized version of biological motion where segments defined by several markers are replaced by one single line, therefore providing less information than other displays. For instance, BM and SK displays reproduce tridimensionality through volumes, depth, and enable the visualization of rotational movement in the transverse plane. The lack of such information in SF may obscure the attribution of expressive properties to the illustrated motion, a task with higher complexity levels than mere motion discrimination.

Regarding expressive condition, results revealed that performances in EXP condition scored significantly higher than in IMO condition. Although sonic variations were imperceptible in the two audio samples used in this study, their ratings diverged considerably. Hence, concomitantly with other studies [67, 68], the visual dimension was the predominant criteria used by participants to classify performances. In the written comments, some participants inclusively mentioned that musicians’ movements potentiate musical experience. Such findings are not surprising, as it is well-known that audiences often grasp expressiveness in music performance based on musicians’ bodily movements rather than musical quality, and that movements with greater amplitude tend to get higher ratings of expressiveness and preferability [4, 30, 31, 36, 69]. Here, we confirmed that this assumption is also applicable to saxophone players, as a first step towards the development of future work involving multiple motion patterns [e.g., 4], instead of focusing on the dichotomy movement versus no movement.

The following limitations were identified in this study. The first relates to the potential confound of the sound by using the two original audio recordings. The original sound was kept to avoid small desynchronizations generated by mismatching audio and motion files. To control this variable, we decided to use audio recordings of the same performer, as it increased similarity in sonic features such as interpretative intention, duration, timbre, articulation, dynamics, ultimately leading to almost no perceivable differences between the movement conditions. In addition, the samples were volume equalized. Nevertheless, the fact that only one performer, one instrument and one musical excerpt were used as stimuli also represents a drawback for the extrapolation of our results. For this study, we intended to focus on the saxophone, as it is part of a larger project centred in this instrument. However, further research including multiple musical pieces and instrumentalists is encouraged. Although online distribution methods are advantageous to reach larger samples, we consider implementing future experiments partially *in loco* to better control participant engagement, apparatus conditions (screen, headphones) and feature adjustments (e.g., lightning, size, volume). Additionally, the inclusion of objective measurements would make the current subjective results more robust and provide useful insights about the physiological reactions to the stimuli. For instance, analysing pupil size as an indicator of attention and arousal [70], gaze direction shifts towards attention-eliciting body locations [68], or even head motion as an involuntary urge to move to the music [71].

An important take-home message emerges from the outcomes of this study about the implications of visual display decisions in future studies, but also in interpreting results from existing ones. According to Hodgins and colleagues [35], given that display types are not equivalent and give rise to different levels of sensitivity of the observer, the most accurate way to evaluate perceptual parameters should be to compare findings from studies complying to the same display standards. This should not be understood as an incentive for adopting the same display in all kinds of perceptual experiment, but rather a recommendation for testing display types before carrying definitive research. The major area of human perception of biological motion requires further research to understand how we grasp, interpret and interact with the world, others and ourselves. Studying it has potential applications for neurodevelopmental diseases [25], individual differences [23], motor learning [51] or interaction in VR environments [32, 43, 44].

## Conclusions

While we found main effects of the factors display and expressive condition isolated in the participants’ ratings, it was the significant interaction effects that allowed to analyse the impact of certain visual displays in moulding ratings across conditions. The displays closer to a human form (mostly SK, sometimes BM) exponentiated the evaluations of expressiveness and music-movement match in the EXP condition, and of overall evaluation in the IMO condition; the opposite trend occurred with the reduced motion display (SF). Hence, greater rating differences were observed between these display types, suggesting displays presenting higher anatomic detail perform better at conveying high-level, subjective properties such as expressiveness or quality than displays providing reduced three-dimensional information. These results underline the relevance of thoroughly considering visual displays as a variable at play in the design of perceptual studies, since they can influence how observers perceive and evaluate human performance. Moreover, we confirmed the assumption that expressive body movement is favored over restricted movement also in the case of saxophone players, the object of our study. This is a preliminary step towards expanding the study to different movement styles and gestures. Exploring how the display types hereby addressed in the context of music performance apply to other visual tasks (e.g., impairment diagnosis, VR interaction) is a potential possibility for future research.

## Supporting information

S1 Video Stimulus used: Projected expressiveness condition, point-light display. (MOV)

S2 Video Stimulus used: Projected expressiveness condition, stick-figure display. (MOV)

S3 Video Stimulus used: Projected expressiveness condition, body mass display. (MOV)

S4 Video Stimulus used: Projected expressiveness condition, skeleton display. (MOV)

S5 Video Stimulus used: Immobile condition, point-light display. (MOV)

S6 Video Stimulus used: Immobile condition, stick-figure display. (MOV)

S7 Video Stimulus used: Immobile condition, body mass display. (MOV)

S8 Video Stimulus used: Immobile condition, skeleton display. (MOV)

S1 Dataset Survey dataset. (XLSX)

S1 File Complementary nonparametric tests. (PDF)
