# Supplementary material for: The impact of visual display of human motion on observers’ perception of music performance
Source: PLoS One. 2023 Mar 8;18(3):e0281755. doi: 10.1371/journal.pone.0281755 (PMC9994732; doi:10.1371/journal.pone.0281755)
Supplement: S1 File — (PDF) [file pone.0281755.s010.pdf]

S10 Supporting Information:
Complementary Nonparametric Tests

We performed Friedman 2-way ANOVAs to support the results of the parametric tests reported in the paper. The level of significance was set at 0.05.

Main Effects of Display

We found significant differences in perceived expressiveness depending on which type of display was adopted for the expressive condition, $\chi^2(3) = 23.212$, $p < 0.001$, and for the immobile condition, $\chi^2(3) = 13.786$, $p = 0.003$.

We also found significant differences in perceived match between music and movement depending on which type of display was adopted for the expressive condition, $\chi^2(3) = 12.338$, $p = 0.006$, but not for the immobile condition, $\chi^2(3) = 3.953$, $p = 0.267$.

Finally, we found significant differences in perceived overall evaluation depending on which type of display was adopted for the expressive condition, $\chi^2(3) = 13.054$, $p = 0.005$, and for the immobile condition, $\chi^2(3) = 33.647$, $p < 0.001$.

Main Effects of Expressive Condition

We found significant differences in perceived expressiveness between the expressive and the immobile condition for the SF, $\chi^2(1) = 121.29$, $p < 0.001$, the PL, $\chi^2(1) = 138.751$, $p < 0.001$, the BM, $\chi^2(1) = 125.776$, $p < 0.001$, and the SK, $\chi^2(1) = 113.255$, $p < 0.001$.

We found significant differences in perceived match between music and movement between the expressive and the immobile condition for the SF, $\chi^2(1) = 163.361$, $p < 0.001$, the PL, $\chi^2(1) = 138.942$, $p < 0.001$, the BM, $\chi^2(1) = 153.806$, $p < 0.001$, and the SK, $\chi^2(1) = 152.824$, $p < 0.001$.

Finally, we found significant differences in perceived overall evaluation between the expressive and the immobile condition for the SF, χ^2 (1) = 121.68, $p < 0.001$, the PL, χ^2 (1) = 131.579, $p < 0.001$, the BM, χ^2 (1) = 149.881, $p < 0.001$, and the SK, χ^2 (1) = 121.68, $p < 0.001$.
